# First report of *Avian metapneumovirus* type B in Iraqi broiler flocks with swollen head syndrome

**DOI:** 10.14202/vetworld.2022.16-21

**Published:** 2022-01-08

**Authors:** Baraa Akeel Al-Hasan, Abdullah O. Alhatami, Husam Muhsen Abdulwahab, Ghadeer Sabah Bustani, Muhammad Ali Hameed, Ameer Haider Jawad

**Affiliations:** 1 Department of Medical Laboratory Technology, College of Medical Technology, The Islamic University, Najaf, Iraq; 2 Department of Microbiology, Faculty of Veterinary Medicine, University of Kufa, Najaf, Iraq; 3 Department of Pathology, Faculty of Veterinary Medicine, University of Kufa, Najaf, Iraq; 4 Department of Physiology and Pharmacology, The Islamic University, Najaf, Iraq; 5 Department of Physiology and Pharmacology, College of Nursing, Altoosi University College, Najaf, Iraq.

**Keywords:** *Avian metapneumovirus* type (B), Iraq, middle Euphrates region, swollen head syndrome, upper respiratory tract infection, viral infection poultry

## Abstract

**Background and Aim:**

Swollen head syndrome (SHS) is a complex disease caused by various agents, including bacterial and viral pathogens, as well as environmental factors. *Avian metapneumovirus* (aMPV) is one of the most important causes of respiratory diseases and SHS in poultry and one of the most widespread viruses worldwide; however, it has not been recorded in Iraq. This study aimed at the molecular identification and subtyping of aMPV in poultry, with the objectives of investigating the prevalence of aMPV in infected broiler flocks with SHS and molecular typing using primers specific to the study of the prevalence of subtypes A, B, and C of aMPV.

**Materials and Methods:**

This study was performed on 67 broiler farms that reported typical SHS from September 2018 to August 2019. Swabs were collected from the trachea, infraorbital sinuses, and lung, then uploaded on FTA cards and subjected to an RNA extraction protocol.

**Results:**

aMPV was detected in 16 (23.8%) samples. Molecular typing using primers specific to the attachment glycoprotein (G) gene showed that all positive samples belonged to subtype B, as assessed using the real-time polymerase chain reaction technique.

**Conclusion:**

aMPV may be the main etiological factor causing SHS in poultry. Moreover, this was the first report of the prevalence of subtype B aMPV strains in broiler farms in Iraq.

## Introduction

*Avian metapneumovirus* (aMPV) is one of the most important etiological factors causing swollen head syndrome (SHS) in poultry. aMPV is a member of the subfamily *Pneumovirinae*, family *Paramyxoviridae*. This family is characterized by an RNA non-segmented [1] and was first isolated in South Africa in 1978 and then reported worldwide [2]; however, it has not been reported in Iraq.

The main economic losses associated with aMPV in layer include a drop in transient egg production, with a high level of egg abnormality. The main clinical signs of this infection are those of a typical respiratory disease, including serous and watery discharge from the nose and ears, which in most cases is followed by frothy tears and conjunctivitis in the late stage of infection. Moreover, SHS will be present because of nostril plugging with a mucopurulent nasal discharge, which is often accompanied by sneezing or coughing and tracheal rales, as well as depression, anorexia, and ruffled feathers in most cases. The incubation period is 3-7 days, and morbidity may reach 100% in birds of all ages. In turn, mortality may vary from 1% to 30% depending on many factors, such as age and constitution of the flock, as well as secondary infections [3].

aMPV causes an acute, highly infectious upper respiratory infection in turkeys. Although young flocks aged 4-9 weeks old are most seriously affected, it may affect all age groups. Moreover, adult poultry are less susceptible to the disease and laying of parental flocks [4]. The upper respiratory tract is primarily affected in young poultry, whereas only a slight respiratory infection with a decline in egg production is observed in laying hens [5]. The typical clinical respiratory symptoms among young flocks of poultry are as follows: Serous watery nasal and ocular discharge; frothy eyes and conjunctivitis; a mucopurulent, turbid nasal discharge and swollen nose later on; and stacky, sneezing, coughing, or tracheal rales. These signs are followed by agitation, anorexia, and rolling feathers [6].

During the first 3-7 days of infection, the virus can spread rapidly among birds in infected flocks within 12-24 h leading to high morbidity which can reach up to 100% [7]. Depending on the age of the flocks and flock composition with secondary infections, mortality can vary between 1% and 30% [4]. For uncontrolled, extreme secondary bacterial infection, up to 90% of the flock may die [8]. In contrast, birds with a good constitution or under experimental conditions without secondary infections may rapidly recover (within 7-10 days of infection). However, with secondary infections, poor management and hygiene will lead to airsacculitis, pericarditis, pneumonia, and perihepatitis, which can prolong and worsen the disease [9].

aMPV can spread horizontally between and within the poultry flock through direct contact or contact with infected objects [10]. This virus has been found to spread rapidly in and through turkey flocks and is, therefore, believed to be highly infectious [11]; in turn, because of its enveloped nature, the virus is quickly deleted after release from many infected hosts to the environment [7]. As aMPV can affect the upper respiratory tract, the main transmission is most likely airborne, particularly through aerosol.

aMPV subtype C was isolated from SPF laying turkey hens with experimentally contaminated eggs up to post-infection (PI) for 7 days [12]; however, Ganapathy *et al*. [13] indicate that the vertical pathway may be short-lived and can play minor role in aMPV transmission.

The previous study regarding the reisolation of aMPV after challenge has proven that infected birds can only release aMPV for a few days [5]. This short shedding period suggests that birds have no latency or carrier status. Convalescent flocks can be reinfected with aMPV throughout fattening because there may be permanent circulation of aMPV within a flock, a farm, or a poultry area. Conversely, convalescent birds were reinfected by aMPV for up to 6 weeks after natural outbreaks [14].

The distribution of aMPV seems to rely on the population density of flocks, as well as the hygiene and biosecurity of poultry [15]. In addition, data suggest that migratory birds may have been involved in the initial migration of the virus from South Africa to European countries [1,16]. In recent years, it has been proposed that aMPV in turkeys in the United States originated from wild bird populations. It was demonstrated that geese, pigeons, and most ducks are refractory to the disease, as do chickens, pheasants, Muscovy ducks, and guinea fowl. An aMPV subtype C RNA was detected in geese, sparrows, and starlings tested in turkey flocks in Minnesota regions of aMPV outbreak [10]. aMPV RNA was also detected in house sparrows, Canada earns, blue-winged teal, and round-billed gulls, as well as sampled snow geese from Saskatchewan, Canada. These results indicate that aMPV transmission does not always occur through close contact between poultry farms and wild birds [16].

When MPV spreads to naive poultry populations, the occurrence of new AMPV infections is very high. Outbreaks of the virus in the UK and Minnesota have been reported [17]. Ironically, the first aMPV epizootic event in Colorado, which infected only a small turkey population, was controlled by slaughter and biosecurity within <1 year following the outbreak. In comparison, the virus circulates for many years with a high frequency and prevalence in the UK and Minnesota [7]. Although the prevalence of AMPV has decreased because of advancements in its understanding, management, and control, AMPV is still present in many countries [11]. A previous study found that, in spring and fall, the seroprevalence of the virus exhibited a seasonal bias with a high incidence. The incidence of seropositive flocks has also been shown to correlate with the regional density of turkey flocks [18]. There was a high prevalence of AMPV in poultry producing areas worldwide; for example, in Germany, Japan, and Israel. A serosurvey study of ostriches tested in Zimbabwe found that 99% of birds were seropositive. A high prevalence of aMPV-specific antibodies, regardless of the current incidence of the SHS, has been shown in all studies [19].

Several experimentalchallenge studies or field experiments have been performed to determine the pathogenesis of aMPV infections; Various factors, such as the clinical outcome, macroscopic and microscopic damage, viral shedding, and the humoral immune response, define the pathogenesis of this disease [18]. More recently, studies have also been carried out that focused on the CMI of aMPV-C [10,20].

Microscopic study of aMPV lesions and viral isolation or the identification of the aMPV genome in infected tissues have shown that cell and tissue tropism in the upper respiratory tract can be confined to the epithelial layer. In layers and breeders, the reproductive tracts may also be infected, as the virus is likely to spread to the respiratory mucosal surface through an aerosol or dust particles in industrial poultry processing [11]. If the virus is on the epithelial layer, the G-protein attachment mediates the connection of the viral particle with the epithelial membrane, and the F-protein subsequently induces the viral envelope to fuse with the hot cell membrane. In addition, the viral genome reaches into the cytoplasm and virus propagation is carried out independently of the nucleus [21]. Fast dissemination and virus shedding increase aMPV propagation not only inside the mucosa of the upper respiratory tract of the infected person but also in the infected turkey flock. That author believed that macrophages that are responsive to aMPV *in vitro* are involved in the systemic propagation of aMPV [22]. During days 2-10 PI, the clinical disease of aMPV was identified, with the greatest incidence and severity of the clinical signs detected between days 5 and 7 PI [5]. The clinical disorder appeared to coincide with viral shedding. Seven PI is isolated from day 1 and up to 10 days in laboratory studies. In chicken, the viral genomes were also detected in choanal swabs for up to 28 days. Based on these studies, it is hypothesized that there is no carrier or latent status and that the time of viral shedding is limited. In addition, the presence and clearance of aMPV in mucosa are hypothesized to correlate with the results and recuperation of microscopic lesions, respectively [23].

This study aimed at the molecular identification and subtyping of aMPV in poultry, with the objectives of investigating the prevalence of aMPV in infected broiler flocks with SHS and molecular typing using primers specific to the study of the prevalence of subtypes A, B, and C of aMPV.

## Materials and Methods

### Ethical approval

The study was approved by Institutional Animal Care and Use Committee, Faculty of Veterinary Medicine, University of Kufa, Iraq (8690-2020).

### Study period, samples collection, and study site

The collection of samples was carried out from the beginning of September 2018 until the end of August 2019 on 67 poultry farms. The ages of the birds ranged between 3 and 6 weeks. Three to four typical SHS cases were collected from each farm and pooled together as one sample, then uploaded onto FTA cards [24]. The fields that were surveyed were distributed in the middle Euphrates region (Baghdad 2, Wasit 10, Karbala 14, Al-Muthanna 7, Al-Najaf 13, and Al-Qadisiyyah 21). The samples were placed on the FTA card to save the genetic materials from microorganisms and for detection using real-time polymerase chain reaction (RT-PCR).

### RNA extraction

RNA was extracted from the FTA cards according to the protocol of Bioneer (Korai) using Accuzol for RNA extraction under aseptic conditions. Subsequently, the extracted genomic RNA was checked using a Nanodrop spectrophotometer, which measures the purity of RNA by reading the absorbance at 260 and 280 nm and calculation the 260/280 nm ratio.

### Detection of aMPV using RT-PCR

aMPV was diagnosed according to the protocol of Thermo Fisher Scientific (USA) using LSI VetMAX aMPV, which is a molecular diagnostic tool for RT-PCR detection of aMPV strains A, B, and C. Each RNA sample obtained after extraction was analyzed in duplex: One well was used for the specific detection of the viral RNA of metapneumovirus strains A and B, and a second well was used for the detection of metapneumovirus strain C and internal positive control (IPC). A positive IPC reflects both the efficiency of the extraction and the absence of inhibitor in the samples. The final volume of the reaction was 25 µL, as shown in Table-1.

**Table 1:** Parts of RT-PCR solution for aMPV detection.

Component	Mix APV A/B	Mix APV A/B Mix APV C/IPC
	
	For 1 reaction	For 1 reaction
1a – Sequences APV A/B	2 µL	-
1b – Sequences APV C/IPC	-	2 µL
2a – Master Mix APV	12.5 µL	12.5 µL
2b – Enzyme APV	0.25 µL	0.25 µL
RNase/DNase-free water	5.25 µL	5.25 µL
Sample	5 µL	5 µL
Total volume	25 µL	25 µL

RT-PCR=Real-time polymerase chain reaction, aMPV=*Avian metapneumovirus*

### Statistical analysis

Data were analyzed and presented using PRISM GraphPad 8 (GraphPad, San Diego, USA), ’numbers application for MAC 11, Statistical Package for the Social Sciences 16.0 (IBM Corp., NY, USA), and Microsoft Excel 2010 (Microsoft, Washington, USA). The obtained data were checked for normal distribution using the Shapiro–Wilk test. A mixed-model analysis of variance (*t*-test and one-way analysis of variance [ANOVA]) was used to compare the differences of means among various groups. The significance was tested using a mixed model (*t*-test and one-way ANOVA); values <0.05 were considered statistically significant. Our data were presented as the standard error of the mean (±SEM).

## Results and Discussion

### Prevalence of aMPV in the middle Euphrates region

**Figure-1:**
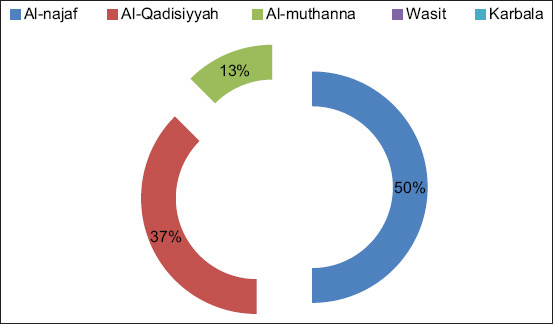
Positive percentage of *Avian metapneumovirus* distribution in middle Euphrates region.

The identification of aMPV was carried out by RT-PCR for the detection aMPV types A, B, and C in the middle Euphrates region. aMPV type B was top of the spot by 16 (23.88%) positive and 51 (76.11%) negative from typical SHS-infected farms, and there was no positive result for other types. The positive results for type B aMPV were distributed over three governorates, that is, Al-Najaf (n=8; 50%), Al-Qadisiyyah (n=6; 37%), and Al-Muthanna (n=2; 13%), whereas Baghdad had no cases of the virus, as shown in Figure-1.

The results obtained for aMPV were expected because of reports of aMPV type B in Iran, Turkey, Jordan, Egypt, and KSA [6,25-27]; therefore, the probability that aMPV entered Iraq because of commercial business with these countries, such as the import of poultry equipment, vaccinations, and eggs, is very high. In turn, migratory birds also play a very important role in aMPV transmission [15].

It has been observed that the prevalence of local aMPV type B was higher than that reported in Iran based on a previous study conducted on turkey [2], but on the other hand the aMPV type B was same as per our results except flocks were vaccinated against aMPV as mentioned in a previous study [6]. In Israel, aMPV type B has a higher prevalence, as reported by our study [18].

### aMPV detection by RT-PCR

**Figure-2:**
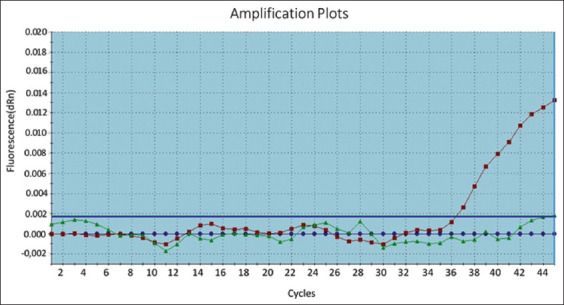
Amplification curve for a positive *Avian metapneumovirus* type B sample with reverse transcription-polymerase chain reaction.

**Figure-3:**
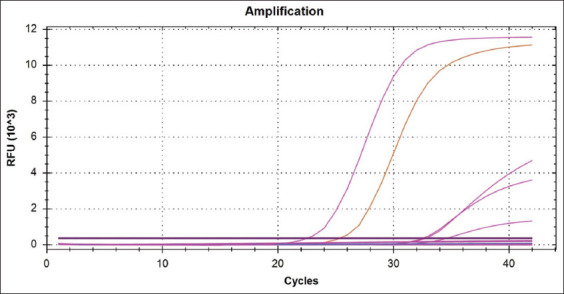
Amplifications curves for *Avian metapneumovirus* type B positive.

aMPV results were based on different filters according to manufacture’s instruction; FAM-TAMRA for aMPV type A and type C, VIC-TAMRA dye for type B aMPV detection. The results were positive aMPV for group of samples Q17, Q18, and Q19 in one well of the FTA card, while type B and were negative for other types, and the CT were 35.0, as shown in Figure-2.On the other hand, the same group that was sent to Ancon for further confirmation of the results yielded the data presented in Table-2 and Figure-3.

**Table 2:** Ancon laboratory results for aMPV type B positive.

Number	Samples	CT for aMPV positive
1	M1, M2	34,1
2	N1, N2, N3, N4	22,1
3	N5, N6, N7, N8	32,1
4	Q1, Q2, Q3	32,3

aMPV=*Avian metapneumovirus*

This method allowed us to detect more than one type of aMPV depending on a previous study that detected more than one type concomitantly. Thus, we suspect that the middle Euphrates region has more than one type of aMPV [28-30]; however, the results were different, as only one aMPV type has been found in the middle Euphrates region and many studies support our results for aMPV type B [30,31]. Moreover, a neutral reference laboratory was used for the confirmation of our results to document aMPV type B.

## Conclusion

This study concluded that aMPV could be implicated as the main etiological factor that causes SHS in poultry. Moreover, this was the first report of the prevalence of the subtype B aMPV strain in broiler farms in Iraq. The aMPV subtypes A and C were not detected in the present study.

## Authors’ Contributions

AOA and HMA: Conceptualized, drafted, and revised the manuscript. BAA and GSB: Collected relevant literature, contributed to the original draft, data curation, investigation, and manuscript review. GSB, MAH, and AHJ: Review of the manuscript and contributed to the editing of the manuscript. All authors read and approved the final manuscript.
